# Comparative Analysis of Magnetic Resonance Spectroscopy and Post-contrast Magnetic Resonance Imaging in the Evaluation of Intracranial Ring-Enhancing Lesions

**DOI:** 10.7759/cureus.114588

**Published:** 2026-08-16

**Authors:** Roopshree Bode, Amit Kumar, Kashi Nath Sarkar, Baidya Nath Singh, Vijendra Ruprela, Harshad Ruprela, Ritika Nihal, Davidson Shandilya, Rajshree Ganjeer, Janganagari Anil

**Affiliations:** 1 Department of Radiodiagnosis, Raipur Institute of Medical Sciences, Raipur, IND

**Keywords:** apparent diffusion coefficient, brain abscess, diffusion-weighted imaging, intracranial ring lesions, magnetic resonance imaging, magnetic resonance spectroscopy, neurocysticercosis, post-contrast mri, ring-enhancing lesions, tuberculoma

## Abstract

Background

Intracranial ring-enhancing lesions are diagnostically heterogeneous because infective, neoplastic, demyelinating, and vascular etiologies may show overlapping conventional magnetic resonance imaging (MRI) appearances. This study evaluated and compared the diagnostic performance of post-contrast MRI and magnetic resonance spectroscopy (MRS) in the etiological characterization of intracranial ring-enhancing lesions.

Materials and methods

This hospital-based prospective observational, non-interventional study included 45 patients with intracranial ring-enhancing lesions. All patients underwent conventional MRI, diffusion-weighted imaging, apparent diffusion coefficient (ADC) assessment, post-contrast imaging, and MRS. Lesion morphology, enhancement pattern, diffusion characteristics, ADC findings, and spectroscopic metabolite patterns were recorded. Final etiological diagnosis was established using a composite reference standard based on histopathology, microbiology, therapeutic response, and clinicoradiological follow-up, wherever available. Categorical variables were summarized as frequencies and percentages. Associations between imaging findings and final diagnosis were assessed statistically, and diagnostic performance was calculated using sensitivity, specificity, positive predictive value, and negative predictive value.

Results

Infective lesions were the most common etiology, accounting for 62.2% of cases, followed by neoplastic lesions in 17.8%, demyelinating lesions in 11.1%, and vascular lesions in 8.9%. Perilesional edema was present in all cases, while mass effect showed a statistically significant association with lesion etiology. Central necrosis and qualitative diffusion restriction were not significantly associated with final diagnosis. ADC assessment showed better diagnostic correlation with lesion etiology. MRS demonstrated stronger diagnostic separation than post-contrast imaging, particularly through elevated choline-based ratios in neoplastic lesions and lipid-lactate patterns in infective lesions. Post-contrast MRI showed sensitivity, specificity, positive predictive value, and negative predictive value of 86.7%, 75.0%, 81.2%, and 80.0%, respectively. MRS showed higher corresponding values of 93.3%, 85.0%, 87.5%, and 91.0%, respectively.

Conclusions

Post-contrast MRI remains essential for anatomical localization and morphological assessment of intracranial ring-enhancing lesions, but its independent diagnostic specificity is limited by overlapping enhancement patterns. MRS provides additional metabolic information and demonstrated superior diagnostic performance in this study. A multiparametric MRI approach combining morphology, diffusion assessment, ADC evaluation, post-contrast imaging, and spectroscopy improves non-invasive etiological characterization and may support more confident clinical decision-making.

## Introduction

Intracranial ring-enhancing lesions represent one of the most frequent and challenging patterns encountered in neuroradiology. Rather than indicating a single disease entity, ring enhancement reflects a shared imaging response to blood-brain barrier disruption, central necrosis, cystic degeneration, abscess formation, granulomatous inflammation, demyelination, or vascular injury. The major etiological categories include pyogenic abscess, tuberculoma, neurocysticercosis, high-grade glioma, metastasis, tumefactive demyelination, subacute infarction, and resolving hematoma [1-4]. This broad differential is clinically important because treatment pathways differ substantially, ranging from antimicrobial therapy and antitubercular treatment to corticosteroids, oncological management, neurosurgical intervention, or conservative follow-up.

Conventional magnetic resonance imaging (MRI) remains the primary imaging modality for detecting and characterizing intracranial ring-enhancing lesions. It provides essential information regarding lesion number, location, size, internal architecture, perilesional edema, mass effect, and enhancement pattern. Post-contrast MRI further improves anatomical assessment by demonstrating rim thickness, regularity, completeness, internal nodularity, and associated meningeal or parenchymal enhancement. Although these morphological features frequently overlap, certain lesion-specific signs can substantially narrow the differential diagnosis. A cystic lesion containing an eccentric mural nodule representing the scolex - the classic “hole-with-dot” sign - is highly characteristic of neurocysticercosis. A smooth thin enhancing ring may suggest an abscess, whereas a thick irregular enhancing wall may favor neoplasm or granulomatous disease, although neither pattern is sufficiently specific in isolation. Similarly, an incomplete or open-ring pattern may favor tumefactive demyelination but should be interpreted in the broader clinical and imaging context [3,4].

Diffusion-weighted imaging (DWI) and apparent diffusion coefficient (ADC) assessment add important physiological information by evaluating water mobility within the lesion core. Restricted diffusion is classically associated with pyogenic abscess because of viscous pus, inflammatory cellularity, and necrotic debris, while necrotic tumors more often demonstrate facilitated diffusion. Nevertheless, overlap persists, as some hypercellular tumors, tuberculomas, and atypical infective lesions may show variable diffusion behavior. Therefore, diffusion findings are valuable but should not be treated as an independent diagnostic endpoint [1,5,6].

Magnetic resonance spectroscopy (MRS) provides metabolic information that complements morphology and diffusion. By assessing metabolites such as choline (Cho), creatine (Cr), N-acetylaspartate (NAA), lactate, lipids, and amino acid peaks, MRS can help distinguish infective, granulomatous, neoplastic, and demyelinating lesions. Elevated Cho and increased Cho-based ratios generally suggest increased membrane turnover, commonly seen in neoplastic lesions, whereas lipid-lactate peaks and amino acid resonance patterns may favor infective etiologies. Tuberculomas often show prominent lipid peaks related to caseation, while pyogenic abscesses may demonstrate characteristic lactate, lipid, acetate, succinate, and amino acid peaks depending on the stage and organism involved [2,5-7].

Despite these advances, no single MRI parameter is universally reliable for etiological classification of intracranial ring-enhancing lesions. Morphological enhancement patterns, diffusion characteristics, and spectroscopic metabolite profiles each contribute different but incomplete diagnostic information. Open-ring enhancement, contrast-enhancement morphology, and broad etiological pattern recognition are useful interpretive clues, but each has important limitations when used alone [8-10].

Previous studies have evaluated DWI and ADC mapping for differentiating brain abscesses, necrotic tumors, cystic tumors, and other intracranial mass lesions [11-14]. Proton MRS has also been studied for characterization of brain abscesses, tuberculomas, neurocysticercosis, and pyogenic intracranial infections [15-22]. Additional literature supports the role of spectroscopy, metabolic imaging, and multiparametric MRI in the evaluation of brain tumors and complex intracranial lesions [23-30].

A multiparametric approach combining conventional MRI, post-contrast assessment, DWI, ADC evaluation, and MRS is therefore more rational than reliance on any one sequence alone. The present study was undertaken to evaluate and compare the diagnostic utility of post-contrast MRI and MRS in the etiological characterization of intracranial ring-enhancing lesions, with additional assessment of diffusion and ADC findings as part of a structured imaging approach.

## Materials and methods

Study design and setting

This was a hospital-based prospective observational, non-interventional, analytical study conducted in the Department of Radiodiagnosis, Raipur Institute of Medical Sciences, Raipur, Chhattisgarh, India. The study evaluated the diagnostic role of post-contrast MRI and MRS in the etiological characterization of intracranial ring-enhancing lesions. The design was observational because no treatment, medication, or therapeutic procedure was assigned by the investigators. Imaging findings were recorded and analyzed systematically after diagnostic MRI evaluation.

Study duration

The study was conducted over an 18-month period from 15 August 2024 to 14 February 2026. The start date was after Institutional Ethics Committee approval dated 14 August 2024. During this period, eligible patients with intracranial ring-enhancing lesions were enrolled consecutively, underwent standardized MRI assessment, and were followed where clinically applicable for final diagnostic correlation.

The study timeline included patient screening, informed consent, MRI acquisition, post-contrast imaging, DWI, ADC assessment, MRS, structured data entry, clinicoradiological correlation, data verification, and statistical analysis.

Ethical approval and consent

The study was approved by the Institutional Ethics Committee, Raipur Institute of Medical Sciences, Raipur, Chhattisgarh, India, approval number IEC/RIMS/2024/01, dated 14 August 2024. Written informed consent was obtained from all participants before inclusion in the study. All patient-related data were anonymized before analysis. No identifying patient information was included in the manuscript, tables, or figures.

Study population and sampling

The study included 45 patients with intracranial ring-enhancing lesions. Patients were enrolled consecutively from those referred for brain MRI and fulfilling the predefined eligibility criteria. Consecutive sampling was used to reduce selection bias and to reflect the clinical spectrum of intracranial ring-enhancing lesions encountered in a tertiary care radiology department.

Inclusion criteria

Patients of all age groups and both sexes were eligible if they had radiologically suspected or confirmed intracranial ring-enhancing lesions and underwent complete MRI evaluation. The required imaging protocol included conventional MRI, DWI, ADC mapping, post-contrast imaging, and MRS. Only patients who provided informed consent were included.

Exclusion criteria

Patients were excluded if they had contraindications to MRI, including non-compatible metallic implants, cardiac pacemakers, retained metallic foreign bodies, or severe claustrophobia. Patients with contraindications to gadolinium-based contrast administration, including known contrast allergy or poor renal function, were also excluded. Additional exclusion criteria included refusal to provide consent, incomplete imaging protocol, non-diagnostic image quality, or ongoing anti-tubercular therapy at the time of imaging.

MRI technique

All examinations were performed using a 1.5 Tesla Siemens MAGNETOM Sempra Elite Edition MRI scanner (Siemens Healthcare GmbH, Erlangen, Germany). A standardized brain MRI protocol was followed. Conventional sequences included T1-weighted imaging, T2-weighted imaging, and fluid-attenuated inversion recovery imaging. DWI and ADC maps were acquired to evaluate diffusion behavior within the lesion core and surrounding tissue.

Post-contrast MRI

Gadolinium-based contrast material was administered at a dose of 0.1 mmol/kg body weight, unless contraindicated. Post-contrast T1-weighted images were evaluated for lesion number, anatomical location, size, ring morphology, wall thickness, wall regularity, completeness of enhancement, internal nodularity, central necrosis, perilesional edema, and mass effect. Enhancement patterns were categorized using predefined morphological criteria as complete or incomplete, smooth or irregular, and thin or thick.

Magnetic resonance spectroscopy (MRS)

MRS was performed using single-voxel spectroscopy and/or multi-voxel chemical shift imaging, depending on lesion size, location, and technical feasibility. Voxel placement was planned to include the most representative part of the lesion while avoiding cerebrospinal fluid spaces, hemorrhage, calcification, and adjacent normal tissue as far as possible. The assessed metabolites included NAA, Cho, Cr, lactate, and lipid peaks. Cho-based ratios, including Cho/Cr and Cho/NAA, were calculated wherever technically feasible.

Image analysis and diagnostic classification

All imaging findings were recorded in a structured data collection sheet. The analyzed variables included age, sex, lesion location, number of lesions, lesion size, perilesional edema, mass effect, central necrosis, post-contrast enhancement pattern, DWI appearance, ADC pattern, and MRS findings.

Final etiological classification was assigned to infective, neoplastic, demyelinating, and vascular categories. The final diagnosis was established using a composite reference standard based on histopathology, microbiology, therapeutic response, and clinicoradiological follow-up, wherever available. This approach was used because histopathological confirmation is not routinely available or ethically required in all intracranial ring-enhancing lesions, particularly when clinical course, laboratory findings, imaging pattern, and therapeutic response support the diagnosis.

Outcome measures

The primary outcome was the diagnostic performance of MRS compared with post-contrast MRI in etiological characterization of intracranial ring-enhancing lesions. Secondary outcomes included the association of lesion morphology, enhancement pattern, diffusion characteristics, ADC findings, and spectroscopic metabolite patterns with final diagnosis.

Statistical analysis

Data were entered into a structured spreadsheet and checked for completeness, coding accuracy, and internal consistency before analysis. Categorical variables were summarized as frequencies and percentages. Continuous variables were assessed for distribution and summarized as mean with standard deviation for normally distributed data and median with range or interquartile range for non-normally distributed data.

Associations between categorical imaging variables and final etiological diagnosis were evaluated using the chi-square test or Fisher’s exact test, as appropriate. Continuous variables were compared using the independent-samples t-test, one-way analysis of variance, Mann-Whitney U test, or Kruskal-Wallis test, depending on data distribution and the number of comparison groups. A two-sided p-value of less than 0.05 was considered statistically significant.

Diagnostic performance of post-contrast MRI and MRS was assessed using sensitivity, specificity, positive predictive value, and negative predictive value against the final etiological diagnosis established using the composite reference standard. The comparative performance of the two imaging approaches was interpreted in relation to this reference standard.

## Results

A total of 45 patients with intracranial ring-enhancing lesions were included in the final analysis. Based on the composite final diagnosis, infective lesions constituted the largest etiological group, comprising 28 cases (62.2%). Neoplastic lesions were identified in eight cases (17.8%), demyelinating lesions in five cases (11.1%), and vascular lesions in four cases (8.9%). The overall demographic, clinical, and etiological profile of the study cohort is summarized in Table 1.

**Table 1:** Baseline Demographic and Clinical Characteristics Stratified by Final Etiological Diagnosis Data are presented as numbers and percentages, n (%), for categorical variables and mean ± standard deviation (SD) for continuous variables. Percentages in each diagnostic category were calculated using the total number of patients within that etiological group as the denominator. Overall percentages were calculated using the full study cohort of 45 patients. Final etiological diagnosis was categorized as infective, neoplastic, demyelinating, or vascular using a composite reference standard based on histopathology, microbiology, therapeutic response, and clinicoradiological follow-up, wherever available. Percentages may not total exactly 100% because of rounding.

Characteristic	Overall	Infective	Neoplastic	Demyelinating	Vascular
Number of patients, n (%)	45 (100.0)	28 (100.0)	8 (100.0)	5 (100.0)	4 (100.0)
Age (years), mean ± SD	37.73 ± 15.85	33.11 ± 14.52	50.38 ± 10.41	47.00 ± 17.29	33.25 ± 18.39
Symptom duration (days), mean ± SD	21.40 ± 10.24	19.18 ± 9.43	24.50 ± 8.35	28.80 ± 14.70	21.50 ± 11.12
Male	31 (68.9)	17 (60.7)	7 (87.5)	4 (80.0)	3 (75.0)
Female	14 (31.1)	11 (39.3)	1 (12.5)	1 (20.0)	1 (25.0)
HIV positive	9 (20.0)	6 (21.4)	1 (12.5)	2 (40.0)	0 (0.0)
Primary malignancy present	0 (0.0)	0 (0.0)	0 (0.0)	0 (0.0)	0 (0.0)
Fever present	24 (53.3)	14 (50.0)	5 (62.5)	3 (60.0)	2 (50.0)
Seizure present	16 (35.6)	10 (35.7)	6 (75.0)	0 (0.0)	0 (0.0)

Lesion characteristics

Lesion characteristics varied across the etiological groups. Lesion size, anatomical distribution, lesion multiplicity, perilesional edema, and mass effect were assessed in all patients. Parietal involvement was the most common lesion location in the overall cohort, followed by temporal, frontal, occipital, and cortical/subcortical locations. Both solitary and multiple lesions were observed, indicating that lesion number alone was not sufficient for etiological differentiation. Perilesional edema was present in all cases, while mass effect was observed in a smaller subset and varied across diagnostic categories. Baseline lesion characteristics according to final etiological diagnosis are shown in Table 2.

**Table 2:** Baseline Lesion Characteristics Stratified by Final Etiological Diagnosis Data are presented as n (%) for categorical variables and mean ± standard deviation (SD) for continuous variables. Percentages in each diagnostic category were calculated using the total number of patients within that etiological group as the denominator; overall percentages were calculated using the full study cohort (N = 45). Location categories were grouped according to the recorded site of the dominant lesion. Solitary indicates a single lesion, and multiple indicates two or more lesions. Percentages may not total exactly 100% because of rounding.

Variable Group	Characteristic	Overall (N = 45)	Infective (n = 28)	Neoplastic (n = 8)	Demyelinating (n = 5)	Vascular (n = 4)
Lesion size	Lesion size (mm), mean ± SD	25.00 ± 12.81	22.24 ± 13.39	31.12 ± 11.38	30.60 ± 9.99	25.00 ± 11.58
Lesion location	Frontal	7 (15.6)	6 (21.4)	0 (0.0)	1 (20.0)	0 (0.0)
	Parietal	17 (37.8)	11 (39.3)	3 (37.5)	3 (60.0)	0 (0.0)
	Temporal	10 (22.2)	6 (21.4)	3 (37.5)	1 (20.0)	0 (0.0)
	Occipital	7 (15.6)	5 (17.9)	2 (25.0)	0 (0.0)	0 (0.0)
	Cortical/subcortical	4 (8.9)	0 (0.0)	0 (0.0)	0 (0.0)	4 (100.0)
Number of lesions	Solitary	25 (55.6)	17 (60.7)	4 (50.0)	2 (40.0)	2 (50.0)
	Multiple	20 (44.4)	11 (39.3)	4 (50.0)	3 (60.0)	2 (50.0)
Associated imaging features	Perilesional edema present	45 (100.0)	28 (100.0)	8 (100.0)	5 (100.0)	4 (100.0)
	Mass effect present	13 (28.9)	4 (14.3)	4 (50.0)	4 (80.0)	1 (25.0)

Conventional MRI, post-contrast imaging, and diffusion findings

Perilesional edema was present in all cases, indicating that edema was a sensitive but non-specific associated imaging feature. Mass effect showed a statistically significant association with final etiological diagnosis, suggesting that the degree of local tissue distortion contributed useful diagnostic information when interpreted with other imaging parameters.

Central necrosis was frequently observed across different etiological groups. However, it did not show a statistically significant association with final diagnosis (p = 0.135), confirming that central necrosis alone was not reliable for differentiating infective, neoplastic, demyelinating, and vascular lesions. Similarly, qualitative diffusion restriction on DWI showed variable distribution across diagnostic categories and did not demonstrate a statistically significant association with final diagnosis (p = 0.262).

In contrast, ADC assessment showed a statistically significant correlation with lesion etiology (p < 0.001). This finding indicates that quantitative diffusion assessment was more useful than qualitative DWI interpretation alone. The combined conventional MRI, post-contrast imaging, DWI, and ADC findings are summarized in Table 3.

**Table 3:** Post-contrast Magnetic Resonance Imaging (MRI), Diffusion-Weighted Imaging (DWI), and Apparent Diffusion Coefficient (ADC) Findings Stratified by Final Etiological Diagnosis Data are presented as numbers and percentages, n (%). Percentages in each diagnostic category were calculated using the total number of patients within that etiological group as the denominator; overall percentages were calculated using the full study cohort (N = 45). Central necrosis was assessed on post-contrast MRI. Diffusion restriction was assessed qualitatively on DWI, and ADC restriction was interpreted on ADC mapping. The p-values refer to the association between each imaging variable and final etiological diagnosis; values were derived using the chi-square test or Fisher’s exact test, as appropriate. Percentages may not total exactly 100% because of rounding.

Variable Group	Imaging Parameter	Category	Overall (N = 45)	Infective (n = 28)	Neoplastic (n = 8)	Demyelinating (n = 5)	Vascular (n = 4)	p-value
Post-contrast MRI	Central necrosis	Absent	2 (4.4)	0 (0.0)	1 (12.5)	1 (20.0)	0 (0.0)	0.135
		Present	43 (95.6)	28 (100.0)	7 (87.5)	4 (80.0)	4 (100.0)	
DWI	Diffusion restriction	Present	17 (37.8)	13 (46.4)	1 (12.5)	1 (20.0)	2 (50.0)	0.262
		Absent	28 (62.2)	15 (53.6)	7 (87.5)	4 (80.0)	2 (50.0)	
ADC mapping	ADC restriction	Present/low ADC	30 (66.7)	25 (89.3)	2 (25.0)	2 (40.0)	1 (25.0)	<0.001
		Absent/high ADC	15 (33.3)	3 (10.7)	6 (75.0)	3 (60.0)	3 (75.0)	

MRS findings

MRS demonstrated distinct metabolic patterns across the major diagnostic categories. Neoplastic lesions more frequently showed elevated Cho and increased Cho-based metabolite ratios, reflecting increased membrane turnover. Infective lesions more commonly demonstrated lipid-lactate peaks, although some overlap was present across categories. Demyelinating lesions showed intermediate metabolic patterns rather than a single uniform spectroscopic profile.

The overall mean ADC value was 0.90 ± 0.33. The mean Cho value was 1.53 ± 0.79, while the mean NAA value was 1.07 ± 0.25. A lipid peak was present in 34 of 45 cases (75.6%) but did not show a significant association with final etiological diagnosis (p = 0.996). Lactate peaks were identified in 12 cases, all within the infective group, and showed a statistically significant association with final diagnosis (p = 0.019), although this finding should not be interpreted as indicating that lactate is specific for infection. Cho values differed significantly among the etiological groups (p = 0.002), while the Cho/Cr and Cho/NAA ratios demonstrated significant intergroup differences (both p < 0.001). The spectroscopic findings according to final etiological diagnosis are presented in Table 4.

**Table 4:** Magnetic Resonance Spectroscopy (MRS) Findings Stratified by Final Etiological Diagnosis Data are presented as numbers and percentages, n (%), for categorical variables and mean ± standard deviation (SD) for continuous variables. Percentages in each diagnostic category were calculated using the total number of patients within that etiological group as the denominator; overall percentages were calculated using the full study cohort (N = 45). MRS variables include metabolite values, lipid and lactate peak patterns, and Cho-based ratios. The p-values for continuous variables were derived using the Kruskal-Wallis test, and p-values for categorical peak patterns were derived using the chi-square test. Percentages may not total exactly 100% because of rounding. NA indicates that statistical comparison was not applicable because the Cr value showed no variability across groups. Cho: choline; Cr: creatine; NAA: N-acetylaspartate; NA: not applicable

Variable Group	Characteristic	Overall (N = 45)	Infective (n = 28)	Neoplastic (n = 8)	Demyelinating (n = 5)	Vascular (n = 4)	p-value
Metabolite values	Cho value, mean ± SD	1.53 ± 0.79	1.25 ± 0.44	2.62 ± 1.06	1.60 ± 0.55	1.25 ± 0.50	0.002
	NAA value, mean ± SD	1.07 ± 0.25	1.04 ± 0.19	1.00 ± 0.00	1.20 ± 0.45	1.25 ± 0.50	0.213
	Cr value, mean ± SD	1.00 ± 0.00	1.00 ± 0.00	1.00 ± 0.00	1.00 ± 0.00	1.00 ± 0.00	NA
Metabolite peak pattern	Lipid peak present	34 (75.6)	21 (75.0)	6 (75.0)	4 (80.0)	3 (75.0)	0.996
	Lipid peak absent	11 (24.4)	7 (25.0)	2 (25.0)	1 (20.0)	1 (25.0)	
	Lactate peak present	12 (26.7)	12 (42.9)	0 (0.0)	0 (0.0)	0 (0.0)	0.019
	Lactate peak absent	33 (73.3)	16 (57.1)	8 (100.0)	5 (100.0)	4 (100.0)	
Metabolite ratios	Cho/Cr ratio, mean ± SD	1.49 ± 0.79	1.21 ± 0.42	2.62 ± 1.06	1.60 ± 0.55	1.00 ± 0.00	<0.001
	Cho/NAA ratio, mean ± SD	1.73 ± 1.30	1.20 ± 0.61	4.05 ± 1.11	1.46 ± 0.77	1.15 ± 0.50	<0.001

Diagnostic performance of post-contrast MRI and MRS

Post-contrast MRI demonstrated moderate diagnostic performance in differentiating intracranial ring-enhancing lesions. It showed sensitivity of 86.7%, specificity of 75.0%, positive predictive value of 81.2%, and negative predictive value of 80.0%. Enhancement patterns provided important morphological information, but overlap was observed between infective and neoplastic lesions, particularly in lesions with thick or irregular ring enhancement.

MRS showed higher diagnostic performance than post-contrast MRI. It demonstrated sensitivity of 93.3%, specificity of 85.0%, positive predictive value of 87.5%, and negative predictive value of 91.0%. The higher negative predictive value of MRS suggests that it was particularly useful in reducing diagnostic uncertainty when conventional and post-contrast findings were overlapping.

The comparative diagnostic performance of post-contrast MRI and MRS is shown in Table 5.

**Table 5:** Diagnostic Performance of Post-contrast Magnetic Resonance Imaging (MRI) and Magnetic Resonance Spectroscopy (MRS) Values are expressed as numerator/denominator and percentage, n/N (%). Diagnostic performance was calculated against the final etiological diagnosis based on a composite reference standard incorporating histopathology, microbiology, therapeutic response, and clinicoradiological follow-up, wherever available. The denominator varies according to the diagnostic-performance parameter. The absolute difference represents the percentage value for MR spectroscopy minus the corresponding percentage value for post-contrast MRI.

Diagnostic Parameter	Post-contrast MRI, n/N (%)	MRS, n/N (%)	Absolute Difference (Percentage Points)
Sensitivity	13/15 (86.7)	14/15 (93.3)	6.6
Specificity	15/20 (75.0)	17/20 (85.0)	10.0
Positive predictive value	13/16 (81.2)	14/16 (87.5)	6.3
Negative predictive value	16/20 (80.0)	20/22 (91.0)	11.0

**Figure 1:**
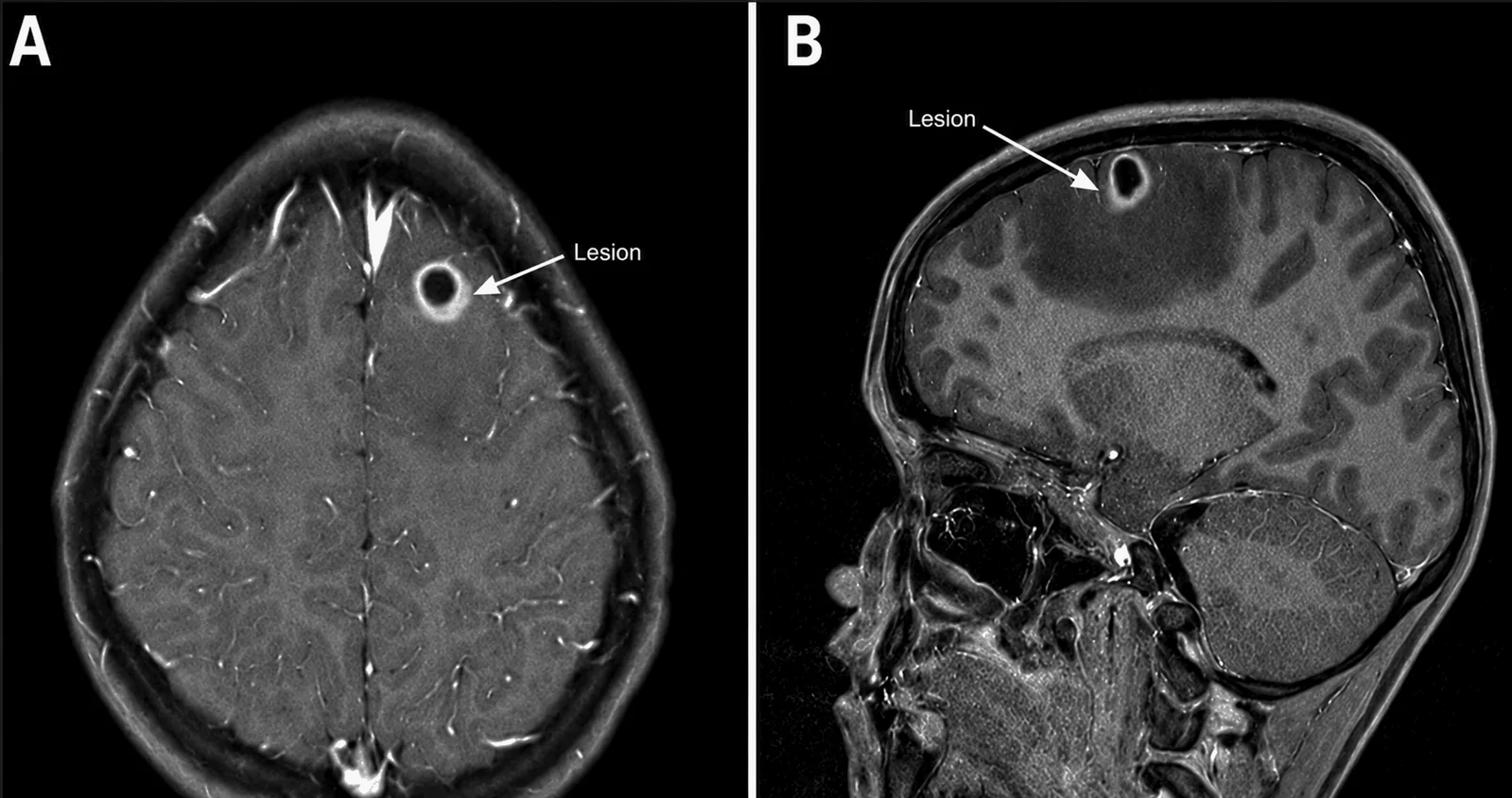
Representative Post-contrast Magnetic Resonance Imaging Appearance of an Intracranial Ring-Enhancing Lesion (A) Axial post-contrast T1-weighted image demonstrates a small ring-enhancing lesion in the cerebral parenchyma, highlighted by the arrow. (B) Sagittal post-contrast T1-weighted image shows the corresponding high frontal convexity ring-enhancing lesion, highlighted by the arrow. These are original anonymized images from the present study. No image has been reused or adapted from a published source. All patient-identifying information has been removed.

Representative post-contrast MRI appearances of an intracranial ring-enhancing lesion are shown in Figure 1.

Overall result summary

In this cohort, post-contrast MRI provided important morphological characterization but had limited independent specificity because of overlapping enhancement patterns. Qualitative DWI findings were not statistically significant when used alone, whereas ADC assessment showed strong diagnostic correlation. MRS provided the strongest diagnostic separation among the evaluated imaging methods, particularly through Cho-based metabolite ratios and lipid-lactate patterns. These findings support the use of a multiparametric MRI approach rather than reliance on a single imaging sequence.

## Discussion

The present study evaluated the diagnostic contribution of post-contrast MRI and MRS in 45 patients with intracranial ring-enhancing lesions. Infective lesions were the dominant etiological group, accounting for 62.2% of cases, followed by neoplastic, demyelinating, and vascular lesions. This distribution reflects the broad diagnostic spectrum of ring-enhancing lesions in routine neuroimaging practice, particularly in settings where infections such as tuberculoma, neurocysticercosis, and abscess remain clinically relevant. Ring enhancement is not a disease-specific sign; it represents a shared imaging endpoint of blood-brain barrier disruption, necrosis, capsule formation, inflammatory response, tumor angiogenesis, or vascular repair. Therefore, interpretation based only on enhancement morphology can be misleading, especially when treatment decisions depend on distinguishing infection from neoplasm or demyelination [1-4,10].

In this study, post-contrast MRI remained essential for anatomical assessment. It clearly demonstrated lesion number, site, wall morphology, central necrosis, perilesional edema, and mass effect. These features are important because they establish the lesion burden and guide the radiologist toward a rational differential diagnosis. However, the diagnostic value of contrast enhancement was limited by overlap among etiological groups. Central necrosis, although frequently encountered, did not show a statistically significant association with final diagnosis (p = 0.135). This is clinically meaningful because necrosis may be seen in abscesses, tuberculomas, high-grade gliomas, metastases, and resolving vascular lesions. Similarly, rim thickness and regularity may suggest a particular diagnosis but rarely provide certainty in isolation. Previous literature has also emphasized that ring enhancement is a pattern-recognition clue rather than a final diagnosis [3,4,8-10].

Perilesional edema was present in all cases, making it a sensitive but non-specific associated finding. Edema reflects local inflammatory response, tumor infiltration, vascular permeability, or tissue injury, and therefore cannot reliably differentiate etiologies when assessed alone. Mass effect, in contrast, showed a statistically significant association with final etiological diagnosis. This suggests that the degree of local tissue distortion adds useful contextual information, particularly when interpreted with lesion size, enhancement pattern, diffusion behavior, and spectroscopy. The lesion characteristics summarized in Table 2 support this observation by showing variation in lesion size, location, multiplicity, edema, and mass effect across diagnostic groups.

DWI is widely used in the evaluation of ring-enhancing lesions because restricted diffusion within a lesion core classically favors pyogenic abscess. The mechanism is related to viscous pus, high cellularity, inflammatory debris, and reduced extracellular water mobility [1,6,11,12,15,20,22]. However, in the present study, qualitative diffusion restriction alone did not show a statistically significant association with final diagnosis (p = 0.262). This finding is important and should not be viewed as contradictory to established radiological teaching. Rather, it highlights a practical limitation: diffusion signal can overlap between abscesses, tuberculomas, hypercellular tumors, and some inflammatory lesions. In addition, visual DWI interpretation may be influenced by T2 shine-through, lesion size, partial volume effect, and heterogeneity within the lesion core [11-14,20,26-29].

ADC assessment showed a stronger statistical association with final etiological diagnosis than qualitative DWI in this cohort (p < 0.001). This finding supports interpretation of DWI together with ADC maps rather than defining restricted diffusion solely on the basis of high DWI signal. However, ADC characteristics should not be regarded as disease-specific. In particular, the relatively low frequency of low ADC within the neoplastic subgroup in this study should not be generalized to all intracranial tumors, because diffusion characteristics may vary substantially with tumor cellularity, histological composition, proportion of viable versus necrotic tissue, and the region sampled for ADC assessment. Highly cellular neoplasms may demonstrate marked diffusion restriction, whereas predominantly necrotic portions of ring-enhancing tumors may show higher ADC values. The combined post-contrast MRI, DWI, and ADC findings are summarized in Table 3. Accordingly, ADC assessment is most useful when interpreted together with enhancement morphology, lesion architecture, spectroscopy, and the clinical context [11-14,20,26-29].

These findings are supported by previous studies showing that DWI and ADC mapping improve differentiation of abscesses from necrotic or cystic tumors, although overlap may occur in tuberculomas, hypercellular tumors, and mixed inflammatory lesions [11-14,20,26-29].

MRS demonstrated greater diagnostic separation than post-contrast morphological assessment within this cohort. Neoplastic lesions showed higher Cho values and Cho-based ratios, consistent with increased membrane turnover and cellular proliferation. Lactate peaks were observed only in the infective subgroup in this study and showed a statistically significant association with final etiological diagnosis; however, lactate is not specific for infection and may also occur in other lesions characterized by anaerobic metabolism or necrosis. In contrast, lipid peaks were common across all etiological groups in the present cohort and did not significantly differentiate them. From a pathophysiological perspective, prominent lipid peaks may occur in caseating tuberculomas, whereas pyogenic abscesses may demonstrate lactate, lipid, amino acid, acetate, or succinate resonances depending on the organism, stage of abscess formation, and technical acquisition factors [2,5,6,15-19,21,22,25]. Accordingly, spectroscopic metabolites should be interpreted as composite metabolic patterns in conjunction with morphology, diffusion characteristics, ADC findings, and clinical context rather than as isolated disease-specific markers.

Prior studies have also shown that proton MRS is useful in brain abscesses, tuberculomas, neurocysticercosis, and other infective or granulomatous lesions by demonstrating lipid, lactate, amino acid, acetate, succinate, and Cho-based metabolite patterns [15-22,25].

The spectroscopic results in this study are consistent with this principle. Cho-based ratios and lipid-lactate patterns showed statistically significant association with final diagnosis (p < 0.001). The overall mean ADC value was 0.90 ± 0.33, while the mean Cho and NAA values were 1.55 ± 0.76 and 1.16 ± 0.24, respectively. Although individual metabolite values should not be interpreted in isolation, their combined pattern was diagnostically useful. The MRS findings are presented in Table 4. These results support the role of spectroscopy as a problem-solving tool when conventional imaging features overlap [2,7,15-24,30].

The diagnostic-performance analysis further strengthens this observation. Post-contrast MRI showed sensitivity of 86.7%, specificity of 75.0%, positive predictive value of 81.2%, and negative predictive value of 80.0%. MRS showed higher values across all four performance measures, with sensitivity of 93.3%, specificity of 85.0%, positive predictive value of 87.5%, and negative predictive value of 91.0%. The absolute improvement was most notable in specificity and negative predictive value. This is clinically relevant because improved specificity may reduce overdiagnosis based on enhancement pattern alone, while a higher negative predictive value may help reduce diagnostic uncertainty when conventional imaging is indeterminate. The comparative performance of both methods is shown in Table 5. Similar diagnostic value of spectroscopy and multiparametric MRI has been reported in previous studies evaluating abscesses, tuberculomas, neurocysticercosis, gliomas, necrotic tumors, and other complex intracranial lesions [15-30].

Similarly, studies on brain tumors and complex intracranial lesions have shown that spectroscopy and multiparametric imaging can improve diagnostic confidence when conventional post-contrast morphology is indeterminate [7,13,14,23,24,28-30].

The representative post-contrast images shown in Figure 1 illustrate a key point of this study: a ring-enhancing lesion can be well demonstrated morphologically, but morphology alone does not define etiology. A small, well-defined enhancing rim with a central non-enhancing component may appear deceptively straightforward, yet similar appearances may be encountered in infection, tumor, demyelination, or vascular lesions. This reinforces the need for a multiparametric approach rather than reliance on a single sequence or imaging sign [8-10,23,24].

The findings of this study are in agreement with prior work showing that combined conventional MRI, diffusion imaging, ADC assessment, and spectroscopy improve confidence in differentiating abscesses, granulomatous lesions, tumors, and pseudotumoral demyelinating lesions [1,2,6-8,11-14,20,23-30]. MRS is particularly useful because it evaluates tissue metabolism rather than only anatomy. However, it is not without limitations. Spectral quality depends on voxel placement, lesion size, magnetic field homogeneity, necrotic content, hemorrhage, calcification, and adjacent cerebrospinal fluid contamination. Small lesions may be technically difficult to assess, and mixed lesions may produce overlapping spectra. For this reason, MRS should not be considered a standalone diagnostic test. Its value is greatest when interpreted with post-contrast morphology, DWI, ADC, clinical background, laboratory findings, therapeutic response, and follow-up [2,7,15-24,30].

This study has several limitations. The overall sample size was modest, with substantial imbalance among the diagnostic subgroups, particularly the relatively small neoplastic, demyelinating, and vascular groups compared with the infective group. This limits the precision of subgroup comparisons and may influence estimates of diagnostic performance. Final diagnosis was based on a composite reference standard rather than uniform histopathological confirmation in every patient; although this reflects routine clinical practice in lesions for which biopsy is not always indicated, it introduces the possibility of verification bias. The study was conducted at a single center, which limits generalizability. Inter-observer agreement was not formally assessed, and therefore the reproducibility of qualitative imaging interpretation between readers could not be quantified. In addition, advanced perfusion and susceptibility-based imaging parameters were not included in the primary diagnostic-performance comparison. Accordingly, the present findings should be interpreted as cohort-specific observations that support the potential complementary value of MRS within a multiparametric MRI approach rather than as definitive estimates applicable to all intracranial ring-enhancing lesions.

Overall, the results indicate that post-contrast MRI remains indispensable for lesion detection and morphological characterization, but MRS adds substantial metabolic information and improves diagnostic confidence. The strongest practical conclusion is not that one sequence should replace another, but that a structured multiparametric approach gives the most reliable diagnostic assessment. In patients with intracranial ring-enhancing lesions, combining morphology, enhancement pattern, diffusion behavior, ADC assessment, and spectroscopy provides a more defensible and clinically useful interpretation than relying on contrast enhancement alone [23-30].

## Conclusions

Post-contrast MRI remains essential for the anatomical evaluation of intracranial ring-enhancing lesions, particularly for assessing lesion location, number, size, rim morphology, perilesional edema, central necrosis, and mass effect. However, enhancement morphology alone has limited specificity because infective, neoplastic, demyelinating, and vascular lesions may show overlapping appearances. Within this study cohort, MRS demonstrated higher sensitivity, specificity, positive predictive value, and negative predictive value than post-contrast MRI. Cho-based metabolite ratios showed significant differences across etiological groups and were higher in the neoplastic subgroup, while lactate was observed predominantly in infective lesions in this cohort; however, these spectroscopic findings should be interpreted as components of an overall metabolic pattern rather than as disease-specific markers. ADC assessment also showed a stronger statistical association with final etiological diagnosis than qualitative DWI alone.

These findings support the complementary use of MRS within a structured multiparametric MRI approach to intracranial ring-enhancing lesions. Integration of conventional morphology, post-contrast enhancement, DWI, ADC assessment, and spectroscopy may improve etiological characterization when interpreted together with clinical and laboratory information. Given the modest sample size, unequal diagnostic subgroups, single-center design, and absence of inter-observer reliability assessment, the diagnostic-performance estimates should be regarded as cohort-specific and require confirmation in larger, preferably multicenter studies. MRS should therefore be considered a complementary problem-solving technique rather than a standalone diagnostic test.

## References

[1] Lai PH, Ho JT, Chen WL, Hsu SS, Wang JS, Pan HB, Yang CF (2002). Brain abscess and necrotic brain tumor: discrimination with proton MR spectroscopy and diffusion-weighted imaging. AJNR Am J Neuroradiol.

[2] Oz G, Alger JR, Barker PB (2014). Clinical proton MR spectroscopy in central nervous system disorders. Radiology.

[3] Reiche W, Schuchardt V, Hagen T, Il'yasov KA, Billmann P, Weber J (2010). Differential diagnosis of intracranial ring enhancing cystic mass lesions - role of diffusion-weighted imaging (DWI) and diffusion-tensor imaging (DTI). Clin Neurol Neurosurg.

[4] Cortese I, Nath A (2006). Case 11: a young woman with ring-enhancing brain lesions. MedGenMed.

[5] Gupta RK, Vatsal DK, Husain N (2001). Differentiation of tuberculous from pyogenic brain abscesses with in vivo proton MR spectroscopy and magnetization transfer MR imaging. AJNR Am J Neuroradiol.

[6] Luthra G, Parihar A, Nath K (2007). Comparative evaluation of fungal, tubercular, and pyogenic brain abscesses with conventional and diffusion MR imaging and proton MR spectroscopy. AJNR Am J Neuroradiol.

[7] Majós C, Aguilera C, Alonso J (2009). Proton MR spectroscopy improves discrimination between tumor and pseudotumoral lesion in solid brain masses. AJNR Am J Neuroradiol.

[8] Masdeu JC, Quinto C, Olivera C, Tenner M, Leslie D, Visintainer P (2000). Open-ring imaging sign: highly specific for atypical brain demyelination. Neurology.

[9] Smirniotopoulos JG, Murphy FM, Rushing EJ, Rees JH, Schroeder JW (2007). Patterns of contrast enhancement in the brain and meninges. Radiographics.

[10] Garg RK, Sinha MK (2010). Multiple ring-enhancing lesions of the brain. J Postgrad Med.

[11] Kim YJ, Chang KH, Song IC, Kim HD, Seong SO, Kim YH, Han MH (1998). Brain abscess and necrotic or cystic brain tumor: discrimination with signal intensity on diffusion-weighted MR imaging. AJR Am J Roentgenol.

[12] Chang SC, Lai PH, Chen WL (2002). Diffusion-weighted MRI features of brain abscess and cystic or necrotic brain tumors: comparison with conventional MRI. Clin Imaging.

[13] Poptani H, Gupta RK, Roy R, Pandey R, Jain VK, Chhabra DK (1995). Characterization of intracranial mass lesions with in vivo proton MR spectroscopy. AJNR Am J Neuroradiol.

[14] Law M, Yang S, Wang H (2003). Glioma grading: sensitivity, specificity, and predictive values of perfusion MR imaging and proton MR spectroscopic imaging compared with conventional MR imaging. AJNR Am J Neuroradiol.

[15] Dev R, Gupta RK, Poptani H, Roy R, Sharma S, Husain M (1998). Role of in vivo proton magnetic resonance spectroscopy in the diagnosis and management of brain abscesses. Neurosurgery.

[16] Burtscher IM, Holtås S (1999). In vivo proton MR spectroscopy of untreated and treated brain abscesses. AJNR Am J Neuroradiol.

[17] Rémy C, Grand S, Laï ES (1995). 1H MRS of human brain abscesses in vivo and in vitro. Magn Reson Med.

[18] Pretell EJ, Martinot C Jr, Garcia HH, Alvarado M, Bustos JA, Martinot C (2005). Differential diagnosis between cerebral tuberculosis and neurocysticercosis by magnetic resonance spectroscopy. J Comput Assist Tomogr.

[19] Gupta RK, Poptani H, Kohli A, Chhabra DK, Sharma B, Gujral RB (1995). In vivo localized proton magnetic resonance spectroscopy of intracranial tuberculomas. Indian J Med Res.

[20] Alam MS, Sajjad Z, Azeemuddin M, Khan ZA, Mubarak F, Akhtar W (2012). Diffusion weighted MR imaging of ring enhancing brain lesions. J Coll Physicians Surg Pak.

[21] Joy L, Sakalecha AK (2023). Role of multiparametric magnetic resonance imaging of the brain in differentiating neurocysticercosis from tuberculoma. Cureus.

[22] Pal D, Bhattacharyya A, Husain M, Prasad KN, Pandey CM, Gupta RK (2010). In vivo proton MR spectroscopy evaluation of pyogenic brain abscesses: a report of 194 cases. AJNR Am J Neuroradiol.

[23] Horská A, Barker PB (2010). Imaging of brain tumors: MR spectroscopy and metabolic imaging. Neuroimaging Clin N Am.

[24] Liserre R, Pinelli L, Gasparotti R (2021). MR spectroscopy in pediatric neuroradiology. Transl Pediatr.

[25] Maheshwarappa RP, Agrawal C, Bansal J (2019). Tuberculoma versus neurocysticercosis: can magnetic resonance spectroscopy and diffusion weighted imaging solve the diagnostic conundrum?. J Clin Diagn Res.

[26] Mishra AM, Gupta RK, Jaggi RS (2004). Role of diffusion-weighted imaging and in vivo proton magnetic resonance spectroscopy in the differential diagnosis of ring-enhancing intracranial cystic mass lesions. J Comput Assist Tomogr.

[27] Stadnik TW, Chaskis C, Michotte A (2001). Diffusion-weighted MR imaging of intracerebral masses: comparison with conventional MR imaging and histologic findings. AJNR Am J Neuroradiol.

[28] Toh CH, Wei KC, Ng SH, Wan YL, Lin CP, Castillo M (2011). Differentiation of brain abscesses from necrotic glioblastomas and cystic metastatic brain tumors with diffusion tensor imaging. AJNR Am J Neuroradiol.

[29] Chiang IC, Hsieh TJ, Chiu ML, Liu GC, Kuo YT, Lin WC (2009). Distinction between pyogenic brain abscess and necrotic brain tumour using 3-tesla MR spectroscopy, diffusion and perfusion imaging. Br J Radiol.

[30] Julià-Sapé M, Acosta D, Majós C (2006). Comparison between neuroimaging classifications and histopathological diagnoses using an international multicenter brain tumor magnetic resonance imaging database. J Neurosurg.

